# Reduced‐toxicity allogeneic hematopoietic stem cell transplantation in congenital sideroblastic anemia

**DOI:** 10.1002/ccr3.1667

**Published:** 2018-08-01

**Authors:** Min Hee Kim, Sanjay Shah, Sylvia S. Bottomley, Niketa C. Shah

**Affiliations:** ^1^ Blood and Marrow Transplantation and Cellular Therapies Children's Hospital of Pittsburgh Pittsburgh PA USA; ^2^ Center for Cancer and Blood Disorders Phoenix Children's Hospital Phoenix AZ USA; ^3^ Department of Medicine Hematology‐Oncology Section University of Oklahoma College of Medicine Oklahoma City OK USA; ^4^ Pediatric Blood and Marrow Transplantation Yale New Haven Children's Hospital New Haven CT USA

**Keywords:** hematopoietic stem cell transplantation, sideroblastic anemia, SLC25A38

## Abstract

The case of an infant girl with severe congenital sideroblastic anemia associated with a novel molecular defect in mitochondrial transporter SLC25A38 is presented. Her transfusion dependence was fully reversed following allogeneic hematopoietic stem cell transplantation using a modified reduced‐intensity conditioning regimen, and she remains healthy 5 years posttransplant.

## 1 INTRODUCTION

Sideroblastic anemia is identified by the unique presence of erythroid precursors in the bone marrow aspirate smear that contain pathologic deposits of iron in mitochondria and are called ring sideroblasts. Occurring as a broad spectrum of erythropoietic disorders, in adults they are most often acquired conditions (eg, in association with a myelodysplastic syndrome, exposure to certain drugs and alcohol, and copper deficiency), while in early life a congenital or inherited sideroblastic anemia (CSA) is most frequent and its molecular basis can be established in over 50% of cases.^1^, ^2^ Recognized defects reside in the pathways of heme synthesis, iron‐sulfur cluster biogenesis, and mitochondrial protein translation. The most common CSA forms are X‐linked sideroblastic anemia due to mutations affecting the erythroid heme synthesis enzyme 5‐aminolevulinate synthase 2 (ALAS2) and the autosomal recessive sideroblastic anemia due to mutations affecting the erythroid‐specific mitochondrial inner membrane protein SLC25A38. Associated ineffective erythropoiesis leads to variably severe anemia and usually systemic iron overload.

For severe, transfusion‐dependent sideroblastic anemia, treatment with hematopoietic stem cell transplantation (HSCT) had been reported in the limited number of six cases with apparent CSA although their molecular basis was not known.^3^, ^4^, ^5^, ^6^ More recently, a few anecdotal cases having identified molecular defects in SLC25A38 who were treated with HSCT were briefly annotated.^7^, ^8^ Here, we describe a patient with CSA associated with novel mutations in the *SLC25A38* gene and her treatment with HSCT using a novel preparative regimen consisting of busulfan, fludarabine, and alemtuzumab with the aim to reduce toxicity.

## 2 CASE REPORT

**Figure 1:**
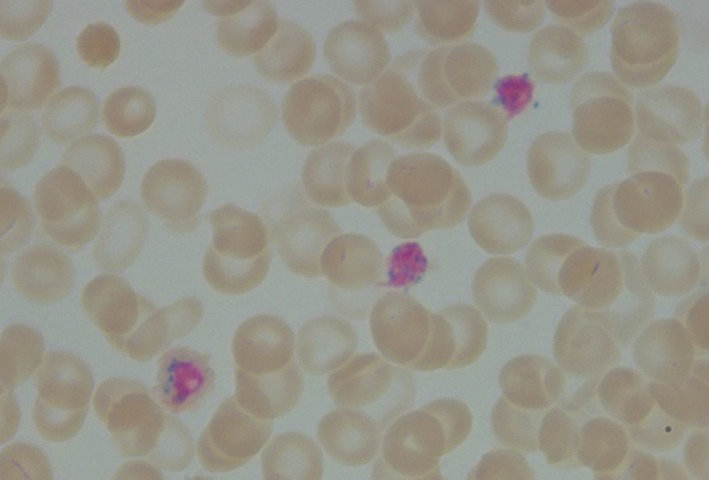
The patient's bone marrow aspirate stained with Prussian blue, showing two ring sideroblasts

**Figure 2:**
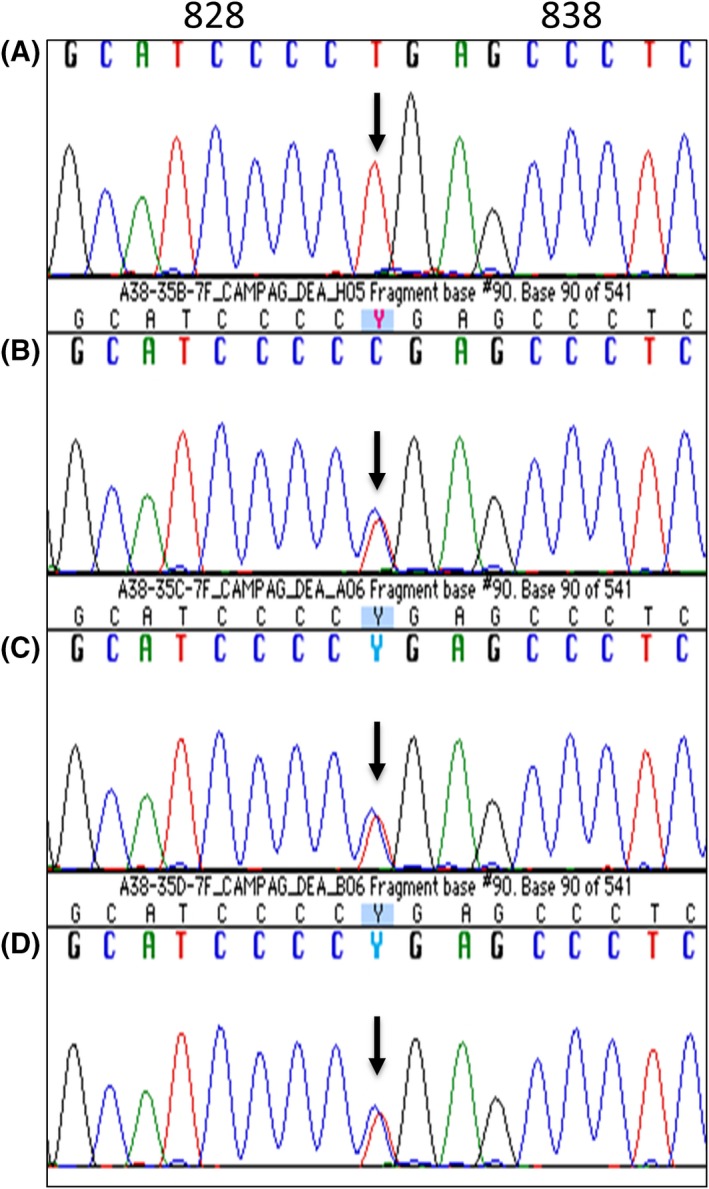
Identification of the mutation in *SLC25A38* by direct sequencing. The patient (A) is homozygous for c.832C>T, and the sister (B), mother (C), and father (D) are heterozygous for the c832C>T change

We present the case of a Hispanic girl who was noted to be pale since birth in 2009 and at 2.5 months of age exhibited severe microcytic anemia with hemoglobin at 3.3 g/dL and mean red cell volume (MCV) of 57 fl; the red blood cell (RBC) relative distribution width (RDW) was 31%, and reticulocytes, 2.3%, and leukocyte and platelet counts were normal. The blood smear showed marked microcytosis, hypochromia, anisocytosis, and nucleated RBCs. At age 5 months, the marrow aspirate revealed mild erythroid hyperplasia and numerous ring sideroblasts (Figure 1). Serum iron data were said to be normal as also erythrocyte protoporphyrins, blood chemistries, and various hematologic studies. Her clinical phenotype was highly suggestive of autosomal recessive CSA. In 2010, Sanger sequencing, targeting the coding and flanking intronic regions of the *SLC25A38* gene,^7^ disclosed a novel homozygous c.832C>T change resulting in a stop codon at arginine 278 (Arg278X) of the protein (Figure 2). Both parents and a sister are carriers (heterozygous) for the mutation. Only distant consanguinity in the family was evident in that the paternal grandmothers of the patient's parents were cousins.

The patient received RBC transfusions every 4‐6 weeks since infancy, and after age 15 months, when the serum ferritin had reached 1700 ng/mL, deferasirox was also administered. At age 4, when the liver iron by MRI was 5.3 mg/g dry tissue and cardiac MRI T2‐star was normal (41.5 ms), she received a 6/6 matched sibling donor bone marrow infusion with a nucleated cell dose of 5.43 × 10^8^/kg. The conditioning regimen consisted of busulfan (1.2 mg/kg every 6 hours for 4 days), fludarabine (40 mg/m^2^/day for 4 days), and an intermediate dose of alemtuzumab (0.2 mg/kg/day for 5 days from day ‐14 to day ‐10). Methotrexate (days +1, +3, and +6) and tacrolimus were given for graft‐vs‐host disease (GVHD) prophylaxis. She tolerated the transplant very well without major complications. Leukocyte engraftment was present on day +17 and a 100% donor chimerism on day +30. RBC transfusion was no longer required after 1 month posttransplant. Subsequently, monthly phlebotomy was performed for 6 months to reduce residual iron overload. At present, nearly 5 years posttransplant, she is healthy with a hemoglobin of 12‐14 g/dL.

## 3 DISCUSSION

Among the nonsyndromic types of CSA so far characterized, the autosomal recessive form due to molecular defects in the glycine transporter SLC25A38 is most common in occurrence after X‐linked sideroblastic anemia.^2^ To date, biallelic mutations in the *SLC25A38* gene associated with CSA have been reported in at least 40 probands or families.^7^, ^8^, ^9^, ^10^, ^11^, ^12^ Most mutations are severe or complete loss‐of‐function mutations. Severe anemia is typically found at birth or in early childhood and requires lifelong transfusions. The burden of supportive care includes iron chelation and avoidance of alloimmunization and infection.

At present, definitive cure for this CSA form may be attempted with allogeneic HSCT, but to date this therapeutic option remains anecdotal.^7^, ^8^ HSCT has become an established treatment for a variety of genetic diseases in childhood, namely thalassemia major, sickle cell anemia, Wiskott‐Aldrich syndrome, Fanconi's constitutional hypoplastic anemia, lysosomal storage diseases, and severe combined immunodeficiency.^13^ The major consideration in this procedure is to provide effective immunosuppression while creating a sufficient degree of donor bone marrow engraftment. Among the previously described six patients with CSA of undefined cause who were treated with HSCT, five received myeloablative conditioning with busulfan and cyclophosphamide +/− antithymocyte globulin (ATG) as the preparative regimen.^3^, ^4^, ^5^ One patient, who could not receive conventional myeloablative conditioning due to underlying comorbidities, received fludarabine, low‐dose total body irradiation, and ATG.^6^ Despite full engraftment, he succumbed to GVHD and prior iron overload on day +190.

The conditioning regimen in our patient, consisting of busulfan, fludarabine, and alemtuzumab, was chosen over traditional myeloablation to decrease transplant‐related toxicities while achieving stable engraftment, which is not seen with very low‐intensity regimens.^14^, ^15^ Fludarabine, a strongly immunosuppressive purine analogue, replaced cyclophosphamide that is known to have increased hepatic toxicity in the presence of busulfan, cardiac toxicity with high doses, and a risk of hemorrhagic cystitis.^16^ Serotherapy with rabbit‐derived antithymocyte globulin (ATG) has been used for many years as prophylaxis for GVHD. However, in recent years it has been replaced by alemtuzumab, a humanized monoclonal antibody directed against CD52, which has had less graft failure and less chronic GVHD in comparison with ATG.^17^, ^18^ In the pediatric age group, single‐center studies using a similar approach have evaluated ATG or alemtuzumab with busulfan and fludarabine for both malignant and nonmalignant diseases. The busulfan/fludarabine/ATG regimen had excellent overall survival; however, graft failure occurred in the majority of children with nonmalignant disorders undergoing mismatched unrelated donor transplants.^18^ The same group reported improved engraftment and decreased GVHD rates using alemtuzumab instead of ATG with busulfan and fludarabine conditioning for malignant and nonmalignant disorders.^19^ Our patient also benefited from this reduced‐toxicity regimen. She continues to have 100% donor chimerism posttransplant without a need for RBC transfusion, signs of iron overload, or GVHD. At nearly 5 years posttransplant, she has no long‐term side effects and is living a healthy life.

In conclusion, this case illustrates that allogeneic bone marrow transplantation using busulfan, fludarabine, and alemtuzumab as the conditioning regimen can be a curative therapy for severe CSA. As the genetic knowledge of CSA becomes extended, we hope to further classify CSAs and tailor each SCT process to aim for continued successful outcomes.

## AUTHORSHIP

MHK, SS, and NCS: involved in patient management. SSB: suggested the diagnosis, facilitated molecular analysis of samples, and provided review and editing of the manuscript. MHK, SS, and NCS: wrote the manuscript.

## CONFLICT OF INTEREST

None declared.
